# Small-Pox in Atlanta

**Published:** 1882-04-20

**Authors:** 


### EDITORIALS AND MISCELLANEOUS.

SMALL-POX IN ATLANTA.—A case of small-pox occurred in Atlanta during the present month. The patient was a negro girl, at one of our Hotels, who probably brought it from a distance. She was promptly quarantined, but six other cases have developed up to this writing, all of which may be traced to the above case.

